# The Relation Between Students’ Theoretical Knowledge and Practical Skills in Endodontics: Retrospective Analysis

**DOI:** 10.2196/46305

**Published:** 2023-04-18

**Authors:** Franziska Haupt, Philipp Kanzow

**Affiliations:** 1 Department of Preventive Dentistry, Periodontology and Cariology University Medical Center Göttingen Göttingen Germany

**Keywords:** curricula, curriculum, dental, dental education, dentist, dentistry, endodontics, endodontology, educational assessment, educational measurement, examination, knowledge assessment, practical skills, skill assessment, theoretical knowledge, undergraduate, undergraduate curriculum, undergraduate education

## Abstract

**Background:**

Dental undergraduate students are required to show sufficient practical skills prior to treating patients. Practical skills and the underlying theoretical knowledge are taught in preclinical courses. Usually, the learning outcome is assessed in written multiple-choice examinations (theoretical knowledge) and practical skills tests. However, students’ assessment of practical skills is more time consuming and prone to bias than objective multiple-choice examinations.

**Objective:**

This study aims to analyze the relation between students’ theoretical knowledge and practical skills in endodontics. Furthermore, the predictive validity of a theoretical knowledge assessment on students’ practical skills was assessed.

**Methods:**

Examination results from all students who participated in the preclinical phantom course in Operative Dentistry (sixth semester of the undergraduate dental curriculum in Germany) between the 2015 summer term and the 2022 summer term were retrospectively evaluated (N=447). The effects of age, sex, previous course participation, and theoretical knowledge on students’ practical skills were assessed, using Pearson correlations, Wilcoxon rank sum tests, and a linear regression analysis. Subsequently, students’ theoretical knowledge and practical skills were compared via a Fisher exact test to identify a suitable pass mark for students’ theoretical knowledge that was associated with sufficient practical skills (≥60%).

**Results:**

Students’ theoretical knowledge was significantly associated with practical skills (*P*_adjusted_=.02; *r*=0.13). By using the current pass mark for theoretical knowledge (ie, 60%), a significant differentiation between insufficient practical skills (<60%) and sufficient practical skills (≥60%) was achieved (*P*=.02). However, for the discrimination between students with sufficient practical skills and students with insufficient practical skills, an adapted pass mark for theoretical knowledge would be more appropriate. The ideal pass mark amounted to 58% (*P*=.02).

**Conclusions:**

Students’ practical skills and theoretical knowledge are significantly correlated. By objectively measuring students’ theoretical knowledge, a rough estimation of students’ practical skills (ie, a differentiation between sufficient and insufficient practical skills) is possible.

## Introduction

Measuring the outcome of education (ie, theoretical knowledge and practical skills) is one of the major issues in dental education. Preclinical teaching within the German undergraduate dental curriculum consists of 6 semesters. During this time, students are taught theoretical knowledge and practical skills for a variety of subjects. Usually, written examinations and practical skills tests are performed to monitor the students’ progress; their capability to apply the acquired knowledge; and, therefore, their ability to treat patients during the subsequent clinical part of the undergraduate dental curriculum. Theoretical knowledge is often objectively assessed via written examinations that use different multiple-choice item types. Practical skills are often assessed by simulating clinical situations that have to be mastered by students. Without any doubt, the implementation of practical skills tests is a complicated process that requires a large amount of time and personnel input [1]. Therefore, several previous studies aimed to assess the correlation between the theoretical knowledge and practical competence of medical students [2-5]. However, only a few studies have evaluated the relation between theoretical knowledge and practical skills among dental students [1,6]. These studies assessed the correlation between students’ achievements in written examinations and objective structured clinical examinations and reported a significant but moderate correlation between both assessments’ scores [1,6].

Ideally, written examinations predict students’ competence and preparedness for further challenges and advanced practice at the end of a preclinical course. Moreover, the measurement process should prevent false-negative results (failing a student who is competent) and false-positive results (passing a student who is incompetent) [7]. Similarly, when applying theoretical knowledge as a predictor for students’ practical skills, an optimal cutoff value has to be calculated, so that the number of false-negative results (theoretically failing but being practically capable) and false-positive ones (theoretically passing but being practically incompetent) is reduced to the greatest possible extent.

In the field of endodontics, both the transfer of theoretical knowledge and the acquisition of basic practical skills play important roles in dental education [8]. A survey regarding undergraduate endodontic teaching among dental schools in the United Kingdom reported that lectures, seminars, tutorials, and laboratory or practical learning were the most frequently applied teaching formats [9]. Furthermore, the *Undergraduate Curriculum Guidelines for Endodontology* of the European Society of Endodontology recommends practical supervision by endodontic specialists or by educators with special interest and training in endodontics [8], which emphasizes the need for practical skills acquisition. However, detailed theoretical knowledge concerning root canal anatomy is a prerequisite for successful endodontic treatment [10]. A survey among undergraduate dental students confirmed the relevance of sufficient anatomical knowledge, as 74% of the students did not feel competent in treating posterior and multirooted teeth with complex anatomies [11]. Moreover, sufficient knowledge about the correct use of endodontic instrumentation systems and their properties, which differ due to the different alloys that these systems are made of, is required for error avoidance during root canal treatment [12]. Without any doubt, theoretical knowledge and the acquisition of practical skills seem to be important factors related to successful endodontic treatment. However, to the best of our knowledge, the correlation between these competencies and the predictive validity of theoretical examinations on the practical capability of dental students in preclinical courses have not been evaluated so far.

This study aimed to analyze the relation between students’ theoretical knowledge and practical skills in endodontics. Furthermore, the predictive validity of a theoretical knowledge assessment on students’ practical skills was assessed, and an optimal cutoff value for theoretical knowledge was defined.

The null hypothesis was that students’ theoretical knowledge does not impact practical skills.

## Methods

### Ethics Approval

This study was approved by the local ethics committee of University Medical Center Göttingen (approval number: 23/10/22). The data analyzed in this study were routinely generated during students’ undergraduate dental education. Participating students did not receive any compensation. The local ethics committee allowed for the secondary analysis of the data set without additional consent. The data set was anonymized prior to this study.

The use of anonymized extracted human teeth in routine teaching practices was approved by the local ethics committee of University Medical Center Göttingen (approval number: 27/8/13). Prior to the collection of extracted teeth during routine care, patients received written information, and informed consent was obtained.

### Participants

All students who were enrolled in the preclinical phantom course in Operative Dentistry (sixth semester of the undergraduate dental curriculum in Germany) between the 2015 summer term and the 2022 summer term were included in the retrospective analysis. Students who did not participate in both the practical skills test and the final written examination (eg, course dropout or absence from examinations due to illness) were excluded.

### Assessment of Theoretical Knowledge

Theoretical knowledge was assessed in summative electronic examinations, using the CAMPUS examination software (Umbrella Consortium for Assessment Networks [13]). Examinations took place at the end of each term and consisted of 30 items. Among these, single-choice items with 5 answer options (Type A items), multiple-select items with 4 to 6 statements (Multiple-True-False items), multiple-select items with 5 to 8 answer options (students were aware of the total number of true answer options that should be selected [Pick-N items]), and open-ended items were used. Single-choice and open-ended items were scored dichotomously (ie, examinees received either 0 or 1 credit point per item). Multiple-True-False items were scored according to the Vorkauf method [14] (in the literature, the terms *Halbpunkt-Bewertung* [14], *Half-point Scoring* [15], and *Vorkauf Method* [16] are used); examinees received 1 credit point if all statements per item were marked correctly, 0.5 credit points if only 1 statement per item was marked incorrectly, and 0 credit points if more than 1 statement per item was marked incorrectly. Pick-N items were scored according to the method proposed by Bauer et al [17] (in the literature, the terms *Partial Scoring 50%* [18] and *PS_50_* [18] are used); examinees received 1 credit point if all true answer options were marked, 0.5 credit points if at least half of the true answer options were marked, and 0 credit points if less than half of the true answer options were marked. Prior to the examinations, all items were reviewed by multiple educators using a checklist for content and formal correctness. The total examination time amounted to 90 seconds per item. A fixed pass mark of 60% was applied.

Each examination covered 3 topics (Cariology/Restorative Dentistry, Endodontics, and Periodontics), but only items on endodontics were considered in this study to allow for a comparison with practical skills in endodontics. Students’ theoretical knowledge was calculated as a relative percentage score based on the number of gained credit points.

### Assessment of Practical Skills

Practical skills were assessed once per term in a standardized practical skills test. Students were given 2.5 hours to perform an endodontic treatment on an extracted human premolar. Teeth were previously embedded in polymethyl methacrylate (Paladur [Kulzer GmbH]) at their physiological position in full-arch models. During the examination, models were mounted in their maxillary or mandibular position and placed in a phantom head (Phantomkopf PK-2 with face mask P-6 GM [Frasaco GmbH]). During the treatment, the use of a rubber dam was mandatory. Assessed treatment steps included (1) the preparation of an endodontic access cavity, (2) the determination of endodontic working length, (3) the preparation of root canals, (4) the cold obturation of root canals by using gutta-percha and sealer, and (5) the cleaning of the endodontic access cavity. Before (preoperative), during (verification of working length), and after the treatments, x-ray images were taken.

Each treatment step was rated by an endodontic specialist using a piloted spreadsheet. For each treatment step, up to 3 raw points were awarded based on students’ performance, and a final practical achievement score was calculated as a relative percentage score. Again, a pass mark of 60% was applied.

### Statistical Analysis

All statistical analyses were performed by using the software R (version 4.2.1; R Foundation for Statistical Computing) [19]. The level of significance was set at an α of .05.

Variables that potentially impacted students’ practical skills (ie, age, sex, previous course participation, and theoretical knowledge) were tested univariately, using Pearson correlations (continuous variables) or Wilcoxon rank sum tests (categorical variables). *P* values were corrected for multiple testing according to the Bonferroni-Holm method. Subsequently, variables were simultaneously entered in a multiple linear regression model.

The number of students with a practical skills level of ≥60% (ie, sufficient) or <60% (ie, insufficient) was determined. By applying the current pass mark of 60% for theoretical knowledge, these students were further categorized as students with a theoretical knowledge mark of <60% or ≥60%. The distribution of students among the four emerging categories was determined by using a Fisher exact test.

To determine the best lower limit for theoretical knowledge, the receiver operator characteristic curve method was applied at 1% intervals. Again, participants were divided into groups of students with a practical skills level of ≥60% or <60%. For the construction of the curve, the number of students with a theoretical knowledge level below a sliding delimiter among both groups was calculated. The Youden index (θ [theoretical knowledge≥delimiter | practical skills≥60%] + θ [theoretical knowledge≥delimiter | practical skills<60%] – 1) and the negative log of Fisher *P* values were used to calculate an optimal theoretical knowledge cutoff value.

## Results

### Participants

A total of 447 students with paired measurements of practical skills and theoretical knowledge were included in this study. Descriptive data and the univariate effect of each variable on students’ practical skills are shown in Table 1. Only the level of theoretical knowledge was significantly associated with practical skills (*P*_adjusted_=.02), indicating a small effect size (*r*=0.13).

The effects of the assessed variables on students’ practical skills were further analyzed via linear regression analysis, as shown in Table 2. The overall model was significant (*F*_4,442_=2.442; *P*=.046; *R*^2^_adjusted_=0.013), indicating a small effect size (*f*^2^=0.01) [20]. Again, only the level of theoretical knowledge was associated with students’ practical skills (*P*=.006). Therefore, the null hypothesis must be rejected.

**Table 1:** Univariate effects of assessed variables on students’ practical skills (practical achievement score).

Variable			Value		Adjusted *P* value^a^	
Age at time of practical skills test (years), mean (SD)			25.06 (3.44)		>.99	
**Sex, n (%)**						.38
	Female		307 (68.7)			
	Male		140 (31.3)			
**Previous course participation, n (%)**						>.99
		No	412 (92.2)			
		Yes	35 (7.8)			
Theoretical knowledge (score), mean (SD)			76.54 (18.5)		.02	

^a^Continuous variables were assessed with *P* values from Pearson correlations. Categorical variables were assessed with *P* values from Wilcoxon rank sum tests.

**Table 2:** Linear regression model^a^.

Variable	Estimate, B	*P* value
Age at time of practical skills test	−0.037	.85
Sex (female vs male)	−1.939	.19
Previous participation (yes vs no)	0.364	.89
Theoretical knowledge	0.102	.006

^a^Intercept=69.971 (*P*<.001).

### Relation Between Theoretical Knowledge and Practical Skills

**Figure 1:**
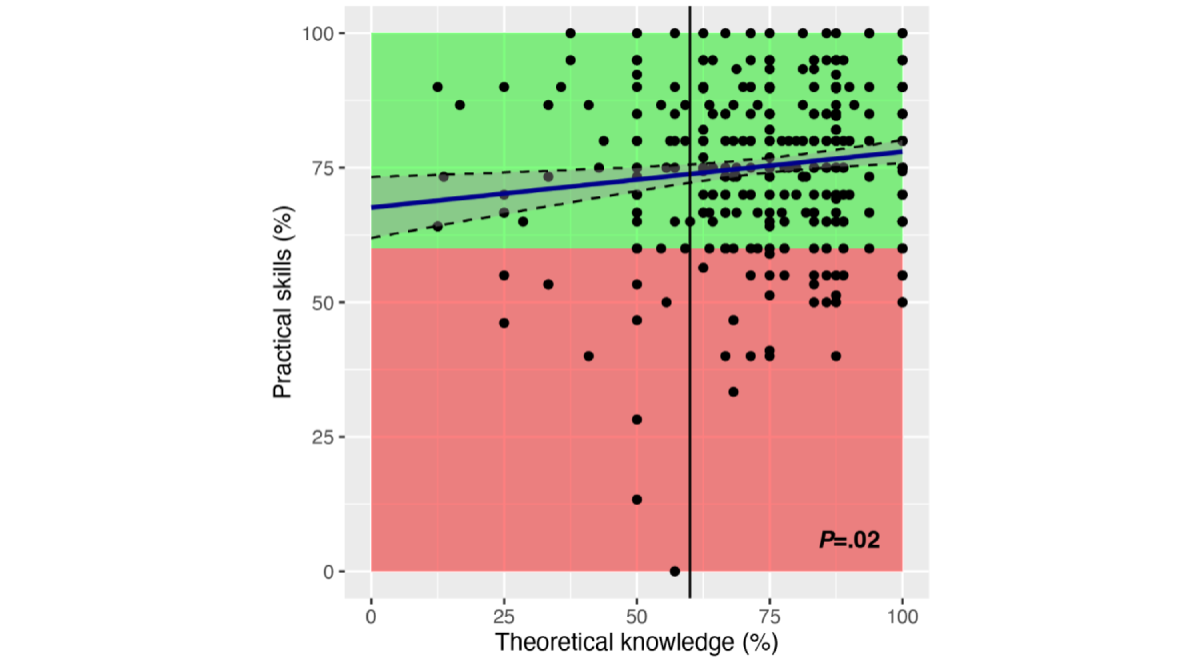
The relation between students’ theoretical knowledge and practical skills. The regression line and 95% CI (dashed lines) are shown. To pass the practical skills test, a minimum practical achievement score of 60% was required (green-shaded area). For the theoretical knowledge assessment in written examinations, a pass mark of 60% was used (vertical line). The *P* value was obtained from a Fisher exact test.

The relation between students’ theoretical knowledge and practical skills is shown in Figure 1. By applying the current pass mark for theoretical knowledge, a significant differentiation was achieved (*P*=.02; Fisher exact test).

### Predictive Validity of Theoretical Knowledge on Practical Skills

**Figure 2:**
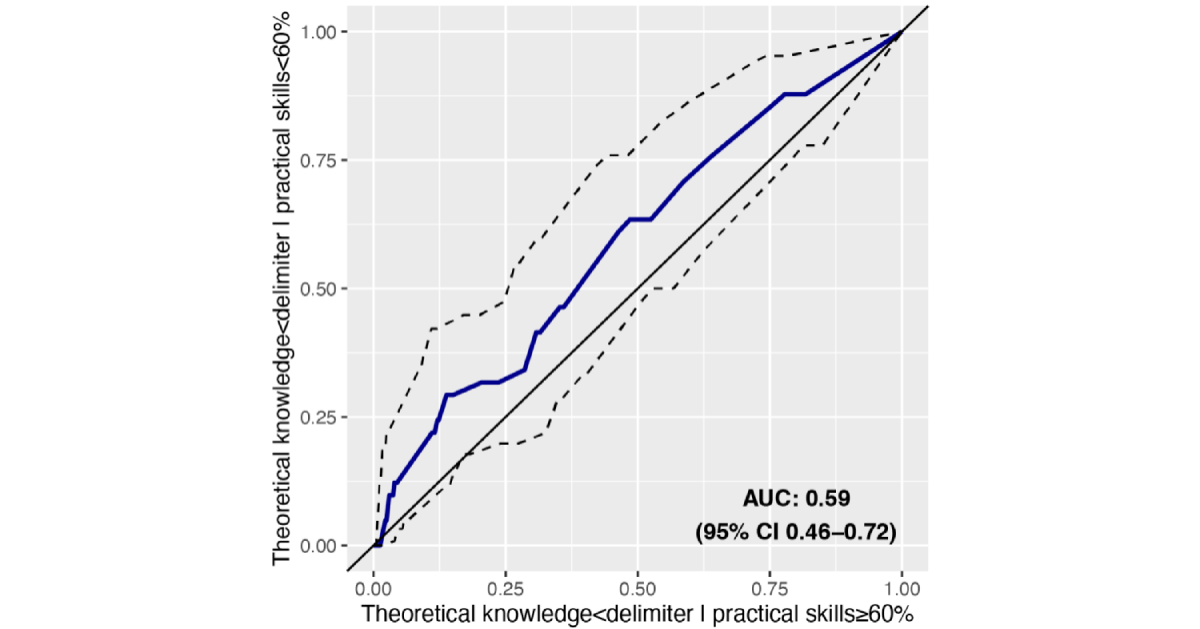
The receiver operator characteristic curve and 95% CI (dashed lines) of theoretical knowledge marks. The putative pass mark for theoretical knowledge was used as a sliding delimiter (0%-100%). AUC: area under the curve.

Based on a fixed pass mark of 60% for practical skills, the area under the receiver operator characteristic curve amounted to 59.2% (95% CI 45.7%-72.1%; Figure 2).

**Figure 3:**
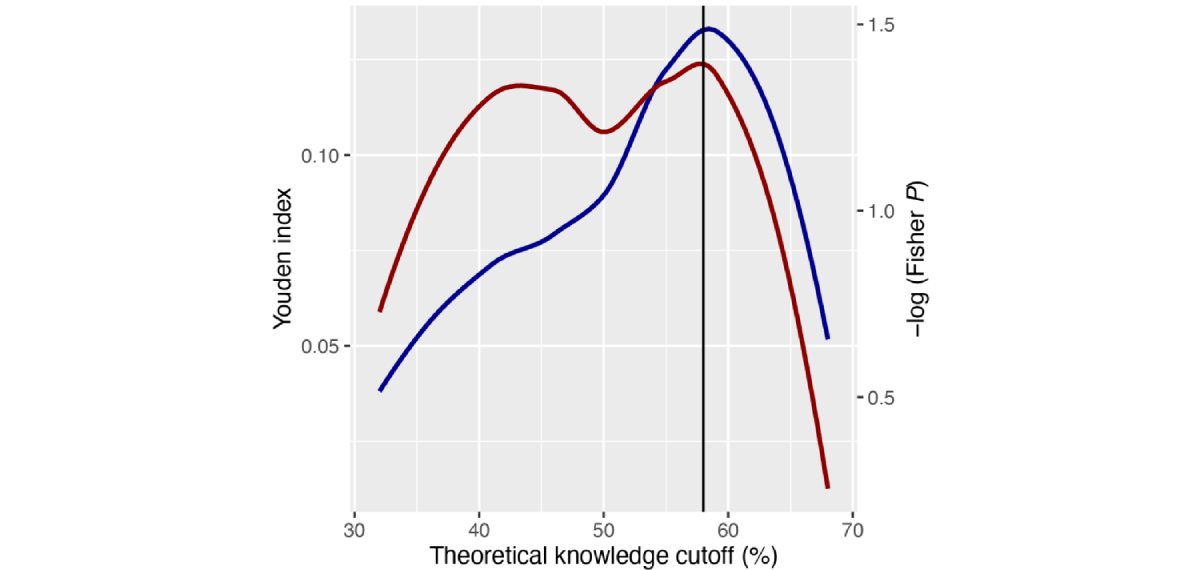
The relation between the Youden index and theoretical knowledge (blue line) and the relation between the negative log of Fisher *P* values and theoretical knowledge (red line) are shown. The putative pass mark for theoretical knowledge was used as a sliding delimiter (0%-100%). Smoothed lines are shown. The maximized Youden index and negative log of Fisher *P* values are indicated by the vertical line.

The best lower limit (ie, pass mark) for theoretical knowledge was 58%, as indicated by the maximized negative log of Fisher *P* values (1.710) and a Youden index of 0.155 (Figure 3). The associated odds ratio amounted to 2.58 (95% CI 1.13-5.59), indicating that students with a theoretical knowledge mark below 58% are 1.22 times more likely to show insufficient practical skills (<60%).

## Discussion

### Principal Findings

This study shows that a significant differentiation between students with sufficient practical skills and students with insufficient practical skills can be achieved by applying the currently used pass mark of 60% on written examinations assessing theoretical knowledge. Nevertheless, it must be mentioned that every test has its limitations and unavoidably results in a specific number of false decisions. Aiming to maximize the number of correctly categorized students (ie, true positives: theoretically passing and being practically capable; true negatives: theoretically failing and being practically incapable), we calculated the best lower limit for theoretical knowledge, which amounted to 58%. Furthermore, the results of this study indicate that students who show insufficient theoretical knowledge (ie, those who achieve a score below 58% in the written multiple-choice examination) are 1.22 times more likely to show insufficient practical skills (ie, achieving a score below 60% in the practical skills test).

### Comparison to Prior Work

The assessment of clinical competence is one of the major issues in medical and dental education. Written examinations, especially those consisting of multiple-choice items, are widely used to objectively assess students’ theoretical knowledge [2,4,6]. However, no single assessment tool that measures all facets of clinical competence has been established yet [21]. The Miller pyramid illustrates several stages of clinical competence, using the terms *knows*, *knows how*, *shows how*, and *does* [22]. Multiple-choice items in written examinations are suitable for assessing both basic facts and applied knowledge (ie, the two lower levels of the Miller pyramid) [21]. Higher levels of clinical competence need to be tested by using practical skills tests or by simulating clinical situations, such as in objective structured clinical examinations [21]. However, according to the Miller pyramid, it is suggested that practical skills are based on sufficient theoretical knowledge. Moreover, the implementation of practical skills tests is cost-intensive, requires many resources, and results in a large amount of personnel effort [1]. Due to these circumstances, previous studies investigated the relationship between the theoretical knowledge and practical skills of medical students [2,4,23-26]. Many of these studies found a significant but weak to moderate correlation between theoretical knowledge and practical skills [1,2,4,6,23,24]. However, some studies failed to show a significant correlation between students’ theoretical knowledge and practical skills [25,26], leading to contradicting results.

Only a few studies have explored the relation between the theoretical knowledge and practical skills of undergraduate dental students [1,6]. Confirming the findings of other previous investigations in medicine, these studies found a moderate correlation between theoretical knowledge and practical performance [1,6]. Similarly, our study shows that theoretical knowledge is significantly associated with practical skills among undergraduate dental students performing root canal treatments (*P*_adjusted_=.02). Two of the major advantages of being able to anticipate the future practical performance of dental students are the possibilities of early intervention and individual promotion. Students who do not perform well on written examinations may benefit from closer monitoring during the early stages of clinical practice. Likewise, students with excellent theoretical performances may be further encouraged and challenged by providing them with more complex cases.

### Strengths and Limitations

The major strengths of this study are the large number of dental students who participated in the preclinical phantom course in Operative Dentistry between the 2015 summer term and the 2022 summer term and the inclusion of student-related variables (ie, age, sex, and previous course participation). However, different limitations are also present. First, the assessed predictive validity of students’ theoretical knowledge on practical skills was based on the used written examinations and practical skills tests. Second, the practical skills tests used extracted human premolars, which potentially could have resulted in inequities. However, all teeth were assessed via x-ray images prior to the practical skills tests, and teeth were excluded if any anatomical difficulties were obvious. Thereby, similar levels of difficulty for the practical skills tests were ensured. The use of extracted human teeth in endodontic skills tests is recommended, as students’ performance on tests involving artificial teeth does not predict their future performance during clinical treatments [27]. Third, the results reflect the competence of undergraduate preclinical course students in performing root canal treatments on extracted teeth of low-level difficulty. Further research is required to assess the relation between the theoretical knowledge and practical competence of more experienced students who treat patients and are confronted with more demanding tasks (ie, during clinical teaching). Fourth, the COVID-19 pandemic occurred in the middle of the study period. However, practical teaching was always fully carried out on site while ensuring sufficient physical distancing (eg, students were placed in 2 cohorts), and theoretical knowledge was partially taught via the internet, as outlined in a previous publication [28]. Thereby, all participating students completed the full practical curriculum, and the pandemic likely did not impact the presented results.

### Future Directions

Although this study found statistically significant results, the weak correlation does not warrant an exact prediction of the practical skills test outcome. Even though the results confirm that the acquisition of sufficient theoretical knowledge is associated with adequate practical skills, the need for the integration of practical courses must be emphasized. Interestingly, the linear regression model of this study shows that previous but unsuccessful participation in the preclinical phantom course had no effect on the outcomes of the practical skills tests when compared to first-time participation in the course. Moreover, 2 previous studies regarding students’ self-perceptions during practical courses reported that most dental students still do not feel confident and competent when performing nonsurgical root canal treatments, especially on premolars and molars [11,29]. This study confirms that theoretical knowledge and extensive practical training (beyond the preclinical course) in endodontics are required to comprehend the importance of each single step in endodontic treatment [30].

### Conclusion

This study provided valuable information concerning the relation between students’ theoretical knowledge and practical skills for performing endodontic treatments. By objectively measuring students’ theoretical knowledge, a rough estimation of students’ practical skills (ie, a differentiation between sufficient and insufficient practical skills) is possible.

## Data Availability

The data sets generated during or analyzed during this study are available from the corresponding author on reasonable request.

## References

[1] Eberhard L, Hassel A, Bäumer A, Becker F, Beck-Mubotter J, Bömicke W, Corcodel N, Cosgarea R, Eiffler C, Giannakopoulos NN, Kraus T, Mahabadi J, Rues S, Schmitter M, Wolff D, Wege KC (2011). Analysis of quality and feasibility of an objective structured clinical examination (OSCE) in preclinical dental education. Eur J Dent Educ.

[2] Auewarakul C, Downing SM, Jaturatamrong U, Praditsuwan R (2005). Sources of validity evidence for an internal medicine student evaluation system: an evaluative study of assessment methods. Med Educ.

[3] Dong T, Saguil A, Artino AR Jr, Gilliland WR, Waechter DM, Lopreaito J, Flanagan A, Durning SJ (2012). Relationship between OSCE scores and other typical medical school performance indicators: a 5-year cohort study. Mil Med.

[4] Eftekhar H, Labaf A, Anvari P, Jamali A, Sheybaee-Moghaddam F (2012). Association of the pre-internship objective structured clinical examination in final year medical students with comprehensive written examinations. Med Educ Online.

[5] Kirton SB, Kravitz L (2011). Objective structured clinical examinations (OSCEs) compared with traditional assessment methods. Am J Pharm Educ.

[6] Ali K, Jerreat M, Zahra D, Tredwin C (2017). Correlations between final-year dental students’ performance on knowledge-based and clinical examinations. J Dent Educ.

[7] van der Vleuten C (2000). Validity of final examinations in undergraduate medical training. BMJ.

[8] De Moor R, Hülsmann M, Kirkevang LL, Tanalp J, Whitworth J (2013). Undergraduate curriculum guidelines for endodontology. Int Endod J.

[9] Al Raisi H, Dummer PMH, Vianna ME (2019). How is endodontics taught? A survey to evaluate undergraduate endodontic teaching in dental schools within the United Kingdom. Int Endod J.

[10] Vertucci FJ (2005). Root canal morphology and its relationship to endodontic procedures. Endod Topics.

[11] Davey J, Bryant ST, Dummer PMH (2015). The confidence of undergraduate dental students when performing root canal treatment and their perception of the quality of endodontic education. Eur J Dent Educ.

[12] Zupanc J, Vahdat-Pajouh N, Schäfer E (2018). New thermomechanically treated NiTi alloys - a review. Int Endod J.

[13] CAMPUS | UCAN ASSESS. Institute for Communication and Assessment Research.

[14] Vorkauf H (1987). Teilpunktbewertung bei K'-Items [Partial credit scoring of Multiple-True-False items]. Jahresbericht 1986 der Gruppe Medizinalprüfungen und der Gruppe Statistik und EDV.

[15] Schmidt D, Raupach T, Wiegand A, Herrmann M, Kanzow P (2021). Relation between examinees’ true knowledge and examination scores: systematic review and exemplary calculations on Multiple-True-False items. Educ Res Rev.

[16] Kanzow P, Schuelper N, Witt D, Wassmann T, Sennhenn-Kirchner S, Wiegand A, Raupach T (2018). Effect of different scoring approaches upon credit assignment when using Multiple True-False items in dental undergraduate examinations. Eur J Dent Educ.

[17] Bauer D, Holzer M, Kopp V, Fischer MR (2011). Pick-N multiple choice-exams: a comparison of scoring algorithms. Adv Health Sci Educ Theory Pract.

[18] Schmidt D, Raupach T, Wiegand A, Herrmann M, Kanzow P (2022). Relation between examinees’ true knowledge and examination scores: systematic review and exemplary calculations on Pick-N items. Educ Res Rev.

[19] The R project for statistical computing. R Foundation for Statistical Computing.

[20] Cohen J (1992). A power primer. Psychol Bull.

[21] Wass V, Van der Vleuten C, Shatzer J, Jones R (2001). Assessment of clinical competence. Lancet.

[22] Miller GE (1990). The assessment of clinical skills/competence/performance. Acad Med.

[23] Sandoval GE, Valenzuela PM, Monge MM, Toso PA, Triviño XC, Wright AC, Paris E, Sánchez I, Valdivia GS (2010). Analysis of a learning assessment system for pediatric internship based upon objective structured clinical examination, clinical practice observation and written examination. J Pediatr (Rio J).

[24] Tijani KH, Giwa SO, Abiola AO, Adesanya AA, Nwawolo CC, Hassan JO (2017). A comparison of the objective structured clinical examination and the traditional oral clinical examination in a Nigerian university. J West Afr Coll Surg.

[25] Schwartz RW, Donnelly MB, Sloan DA, Johnson SB, Strodel WE (1995). The relationship between faculty ward evaluations, OSCE, and ABSITE as measures of surgical intern performance. Am J Surg.

[26] Johnson G, Reynard K (1994). Assessment of an objective structured clinical examination (OSCE) for undergraduate students in accident and emergency medicine. J Accid Emerg Med.

[27] Bitter K, Gruner D, Wolf O, Schwendicke F (2016). Artificial versus natural teeth for preclinical endodontic training: A randomized controlled trial. J Endod.

[28] Kanzow P, Krantz-Schäfers C, Hülsmann M (2021). Remote teaching in a preclinical phantom course in Operative Dentistry during the COVID-19 pandemic: Observational case study. JMIR Med Educ.

[29] Murray CM, Chandler NP (2014). Undergraduate endodontic teaching in New Zealand: students’ experience, perceptions and self-confidence levels. Aust Endod J.

[30] Picart G, Pouhaër M, Dautel A, Pérard M, Le Clerc J (2022). Dental students’ observations about teaching of endodontic access cavities in a French dental school. Eur J Dent Educ.

